# Cytokinetic Abscission Regulation in Neural Stem Cells and Tissue Development

**DOI:** 10.1007/s40778-021-00193-7

**Published:** 2021-08-11

**Authors:** Katrina C. McNeely, Noelle D. Dwyer

**Affiliations:** 1 Department of Cell Biology, University of Virginia School of Medicine, PO Box 800732, Charlottesville, VA, USA

**Keywords:** Cytokinesis, Abscission, Neural stem cells, Cell fate, Microcephaly, Midbody

## Abstract

**Purpose of Review:**

How stem cells balance proliferation with differentiation, giving rise to specific daughter cells during development to build an embryo or tissue, remains an open question. Here, we discuss recent evidence that cytokinetic abscission regulation in stem cells, particularly neural stem cells (NSCs), is part of the answer. Abscission is a multi-step process mediated by the midbody, a microtubule-based structure formed in the intercellular bridge between daughter cells after mitosis.

**Recent Findings:**

Human mutations and mouse knockouts in abscission genes reveal that subtle disruptions of NSC abscission can cause brain malformations. Experiments in several epithelial systems have shown that midbodies serve as scaffolds for apical junction proteins and are positioned near apical membrane fate determinants. Abscission timing is tightly controlled and developmentally regulated in stem cells, with delayed abscission in early embryos and faster abscission later. Midbody remnants (MBRs) contain over 400 proteins and may influence polarity, fate, and ciliogenesis.

**Summary:**

As NSCs and other stem cells build tissues, they tightly regulate three aspects of abscission: midbody positioning, duration, and MBR handling. Midbody positioning and remnants establish or maintain cell polarity. MBRs are deposited on the apical membranes of epithelia, can be released or internalized by surrounding cells, and may sequester fate determinants or transfer information between cells. Work in cell lines and simpler systems has shown multiple roles for abscission regulation influencing stem cell polarity, potency, and daughter fates during development. Elucidating how the abscission process influences cell fate and tissue growth is important for our continued understanding of brain development and stem cell biology.

## Introduction

**Fig. 1:**
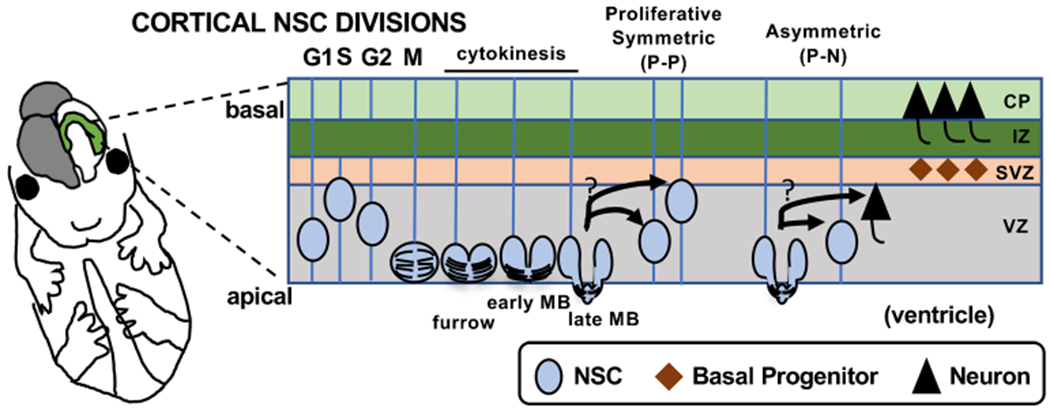
Embryonic cortical NSC modes of division and daughter cell types. Schematic of cross section of embryonic mouse cerebral cortex shows a neural stem cell (NSC) undergoing mitosis (M) and polarized cytokinesis. The midbody (MB) forms at the apical membrane and mediates abscission. See text for more details. cp, cortical plate; iz, intermediate zone; svz, subventricular zone; vz, ventricular zone

Proper development of the mammalian brain requires a series of coordinated cell divisions to produce tremendous numbers of neurons and glia at the correct times. Embryonic neural stem cells (NSCs) reside in a polarized epithelium and have a highly polarized structure that extends from the basal lamina to the ventricular surface. The apical membranes (apical endfeet) are joined by adhesive junctions and make up the lateral ventricle walls. The NSC nuclei migrate within the cell during the cell cycle: basally before S-phase and apically to the ventricular surface for mitosis (Figure 1). Early in development (~ embryonic day (E) 8–E11 in mouse), NSCs expand the pool of stem cells through proliferative symmetric division. This increases the area of the neuroepithelial sheet. Later (~E12), NSCs begin to gradually switch to asymmetric divisions, to start making neurons. Neurons are post-mitotic cells that do not divide again. They migrate away from the ventricle to form the cortical plate (cp, neuronal layer). Thus, neurogenesis increases the thickness of the brain [1]. How NSCs regulate these modes of division to create the correct number and types of daughter cells and make a brain of the correct size and structure is an intense area of study. This review will discuss recent literature suggesting that regulation of cytokinetic abscission plays a role in this process.

NSCs undergo a polarized form of cytokinesis that may be important for maintaining their stemness, as well as their polarity and epithelium integrity. These tall thin cells must split their organelles, cytoplasm, membrane, and apical cell junctions into two daughter cells of equal or unequal fates. Cytokinesis consists of two distinct steps: cleavage furrowing and abscission. The cleavage furrow ingresses from basal to apical, forming an intercellular bridge at the apical membrane (Figure 1). The intercellular bridge contains the compacted antiparallel microtubules of the central spindle, which form the midbody (MB). The midbody serves as a platform that mediates the process of abscission, severing the intercellular bridge.

**Fig. 2:**
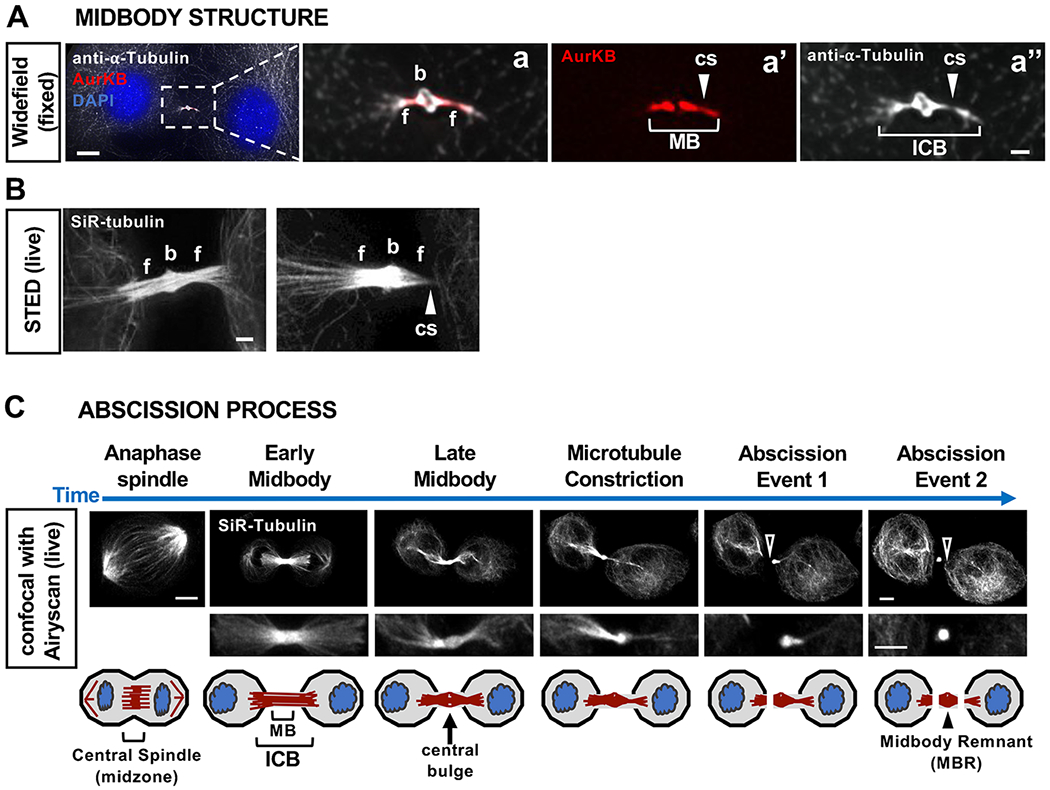
Midbody subdomains and abscission process. (A) Widefield image of a mouse embryonic fibroblast at a late stage of abscission, fixed and immunostained for alpha-tubulin (white) and Aurora B kinase (AurKB, red, MB flanks). ICB, intercellular bridge; b, bulge; f, flank; cs, constriction site. (B) The microtubule organization of midbodies is revealed by labeling live HeLa cells with SiR-Tubulin, and stimulated emission depletion (STED) microscopy. (C) Steps in the abscission process visualized in live cells with SiR-tubulin. The midbody is formed by the compaction of central spindle microtubules, then matures, gradually becoming thinner with a central bulge and constriction sites. Microtubules are disassembled to sever each flank (arrowhead), completing the separation of daughter cells and releasing the MBR extracellularly. HeLa cells were imaged by time-lapse confocal microscopy with Airyscan. Scale bars: 1 μm in Aa-Aa”,B; 5 μm in A,C. See [36] for methods

Much of our knowledge about the mechanisms of abscission comes from studies in cell lines or single cell systems. Abscission takes much longer than cleavage furrowing, completing in G1 phase of the next cell cycle in HeLa cells [2]. The midbody is comprised of over 400 proteins [3••, 4] that assemble within two major subdomains: a central bulge with an electron-dense core, sometimes called the Flemming body, and flanks on each side (Figure 2A, B) [5, 6]. As the midbody matures, a constriction site (cs) will form on each flank, with local thinning where severing will occur. Midbody severing involves both local disassembly of the cytoskeleton (microtubules, actin, and septins) and scission of the plasma membrane (for review, see [7, 8]). Microtubule disassembly happens concurrently with membrane scission, both thought to be mediated by endosomal sorting complex required for transport (ESCRT) machinery [9, 10]. These sequential steps in the process of abscission can be visualized by observing changes in the midbody microtubule organization (Figure 2C). After abscission, the central bulge remains intact and is known as the midbody remnant (MBR). In cells where both midbody flanks are severed, the MBR is released extracellularly. It may remain on the cell surface, or be internalized by a daughter cell or other nearby cell. Several roles for MBRs have been proposed to transmit signals to neighboring cells through surface binding or internalization [11–15] including influencing stemness or differentiation [16, 17]

Work from the past decade provides increasing evidence that abscission regulation plays roles in many developmental processes, including cell fate determination, polarization, and tissue morphogenesis. This review will focus on the importance of abscission regulation in neural stem cells for proper mammalian brain development. Strong evidence is provided by mutations in abscission genes in both mice and humans that cause brain malformations. Analyses of abscission defects in these mouse mutants, as well as data from simpler systems, suggest that three particular aspects of abscission must be tightly regulated by NSCs as they build the brain: midbody positioning, abscission timing, and midbody remnant handling. In vivo studies of the mechanisms and roles of abscission in stem cells and tissue development have only just begun, and many questions remain.

## Mouse and Human Mutations Reveal Dire Consequences of Abscission Dysregulation in Neural Stem Cells

Brain development is particularly vulnerable to defects in cytokinetic abscission. This is made evident by human and mouse mutations of abscission genes that cause forms of microcephaly: *Cep55, Kif20A, Kif20B, Kif14, CitK*, and *Sept7* [18–35]. With the exception of *Sept7*, all of these mutations affect brain growth more severely than that of other tissues. Interestingly, two of these genes, *Cep55* and *Kif20B*, are not present in invertebrate genomes, suggesting they may have evolved to help build bigger more complex nervous systems. While there are other cytokinesis genes expressed in NSCs, we focus on these six because they have relatively specific roles in abscission as defined in cell line studies, and the mouse knockouts have documented defects in cortical development (see Table 1).

The proteins encoded by these genes all localize to the midbody and play different roles in regulating abscission. These roles were first defined by studies in mammalian cell lines. Two related kinesin motor proteins of the Kinesin-6 family, Kif20A (also called MKLP2) and Kif20B, localize on the midbody flanks, but with slightly different spatiotemporal patterns [28, 36]. Kif20B appears to be important for tight bundling of midbody microtubules, while Kif20A is required to localize Aurora B kinase to the midbody, and both appear to regulate abscission timing [36–38]. Kif20A has been shown to interact with a midbody flank protein, Septin 7 (Sept7) [23]. Septins are GTP-binding proteins that form filaments at the cell cortex or with other cytoskeletal proteins. Sept7 localizes to the central spindle during furrowing and to the central bulge of the midbody during abscission, and is believed to be important for both the maintenance of the furrow and microtubule disassembly at abscission sites [35, 39, 40]. Kif14 (of the Kinesin-3 family) is required to localize Citron Kinase (CitK) to the central spindle and central bulge of the midbody [41]. CitK regulates abscission timing and helps to maintain midbody stability [42]. Cep55 is a coiled-coil protein that forms a disc at the middle of the central bulge, and ensures efficient recruitment of TSG101 and Alix, which then recruit the ESCRT-III components that mediate scission of the midbody (for review, see [43]).

While studying cytokinesis gene functions in cell lines helps to understand how they cause disease, studying the in vivo phenotypes of humans and mice carrying mutations is necessary to elucidate the mechanisms and roles of abscission regulation in stem cells and development. Cell line studies in two-dimensional culture cannot model certain aspects of stem cell biology such as three-dimensional structures, or giving rise to different daughter cell types at different times in development. Perhaps not surprisingly, all six of the abscission genes we are discussing have been associated with human cancers. Thus far, three of them, *CEP55, KIF14*, and *CITK*, have been associated with specific human microcephaly syndromes (Table 1). Depending on the specific mutation, lethal or non-lethal syndromes affecting development of the brain and other organs can result. A common feature of patients with non-lethal disease is intellectual and developmental delay (ID/DD). The lethal syndromes often affect development of both the kidney and cerebellum. To probe the roles of these and other cytokinesis genes in brain development, we depend primarily on mouse models.

The gross phenotypes of the mouse mutants in these abscission genes share some features in common (Table 1). Perhaps unexpectedly, the homozygous knockouts, with the exception of *Sept7*, are able to develop fairly normally up until birth, but die soon after [19, 21, 24, 25, 28, 29]. Interestingly, *Sept7* can only be studied as a conditional mutant because the null embryos die between E7 and E10 [23, 35]. Two mutants have a noticeably smaller body at birth (*Kif20B* and *Kif20A*), while three have almost normal body size when born, but then fail to thrive postnatally (*Cep55*, *Kif14*, and *CitK*). Three of the mutants have flat foreheads (*Cep55*, *CitK*, and *Kif14*), and one has a short snout and small eyes (*Kif20B*).

Knockout mice of all of these abscission genes have small cerebral cortices (microcephaly). Remarkably, most have preserved layer structure, even though layers are thinner. The exception is the *Kif14* mutant, which has superficial layer neurons present in deep layers, suggesting a possible neuron migration defect [25]. Interestingly, all of the flat-headed mutants have ataxia, which could be caused by the observed defects in cerebellum development.

What are the consequences for the daughter cells of abnormal NSC abscissions that account for the deficits in brain growth? Prior work in cell lines in vitro showed that knockdown of abscission genes could cause daughter cells to become binucleate or have persistent intercellular bridges. However, in vivo, binucleation is not necessarily a hallmark of these mouse abscission mutants. *Cep55* and *CitK* mutations do result in binucleate cells in both the mouse and human brains [19, 29], but *Kif20A, Kif20B*, and *Sept7* mutations do not [21, 23, 28]. For *KIF14* mutations, there are binucleate cells in human patients [26], but it was not examined in the mice. It is not clear if the discrepancies between in vitro and in vivo results are due to compensation, off-target knockdowns, or differences between 2-D cultures and 3-D tissues. Another differing outcome from gene loss in cell lines versus in vivo is in regard to apoptosis. Immortalized or cancer cell lines do not have normal regulation of apoptosis, although it is sometimes reported after long delays in abscission failure [44, 45]. However, there is elevated apoptosis in the brains of all of these mouse mutants [19, 21, 23–25, 28, 29, 46, 47]. This apoptosis was determined to be p53-dependent in *Cep55, Kif20B*, and *CitK* mutants. In the *Cep55* knockout, p53 elevation was shown to correlate with binucleation in NSCs [29•]. Importantly, NSCs appear to have a lower threshold for p53 activation than other cell types [29•, 46, 48]. A third consequence of abscission dysregulation that is different in vitro versus in vivo is cell cycle arrest versus cell cycle exit for differentiation. Some cell lines such as RPE1 cells can exhibit cell cycle arrest after failures of cytokinesis, but in vivo, cells that fail cytokinesis can differentiate. During normal embryonic brain development, the daughter cells of NSC divisions that commit to a neuron cell fate exit the cell cycle and terminally differentiate [49]. In *Cep55, Kif20A*, and *Sept7* mutants, there are excess neuron progeny at early ages (premature neurogenesis). Additionally, individual NSC divisions were analyzed in these mouse mutants. All of them have an increase in neurogenic divisions at the cost of proliferative symmetric divisions, meaning that more daughter cells exit the cell cycle to differentiate into neurons [23, 28, 29•]. Unlike apoptosis, the increased neurogenesis is not dependent on p53, at least in the *Cep55* knockout [29•]. Together these data suggest that disruption of abscission regulation can cause microcephaly by depleting stem cells, either directly or indirectly, through apoptosis and premature neurogenesis, perhaps sometimes after binucleation.

The amount of binucleation, apoptosis, and premature neurogenesis vary in these different gene knockouts. What are the precise abscission defects in NSCs caused by loss of these abscission genes? In the cases where the knockout studies did not specifically analyze cytokinesis regulation during brain development, the direct roles these proteins play in epithelial NSC abscission are not clear. In the remainder of this review, we will focus on the mouse mutants in *Cep55* and *Kif20B*, for which specific roles in NSC abscission were identified, as well as informative perturbations of abscission in simpler model systems. We will discuss three particular aspects of abscission that these studies have identified as important for stem cell, epithelial, and brain development: midbody position, abscission duration, and MBR disposal.

## Midbody Positioning in Cell Division Can Provide Polarity Cues to Daughter Cells

In many dividing cells, remnants of cytokinetic abscission provide polarity cues for daughter cells. Budding yeasts do not have a midbody, but the site of abscission from the previous division, the bud scar, determines where the next bud will form [50]. In the *C. elegans* zygote, the midbody remnant from the first cell division is internalized by the posterior daughter cell, moves ventrally, and thereby helps establish dorsal-ventral polarity within the embryo [15]. Newborn neurons in *C. elegans* and *Drosophila* appear to use cytokinesis remnants to establish an apical pole where the first neurite grows [51, 52]

## Midbody Positioning Is Associated with Apical Membrane and Apical Junctions in Polarized Epithelia

Accumulating evidence suggests that MB and MBR positioning contributes to apical membrane polarity and lumen formation set-up in early development. When Madin-Darby Canine Kidney (MDCK) cells are grown in 3-D culture, a cyst-like structure will form with an apical membrane facing a fluid-filled lumen. In this system, lumen formation is initiated by the assembly of an “apical membrane initiation site” at the midbody, and maintained by consistent MB positioning at the apical membrane [53, 54]. After abscission, the MBRs remain at the MDCK apical membrane; but, if they are experimentally displaced, ectopic lumen formation occurs [55]. The strongest evidence for midbody positioning inducing apical polarity comes from in vivo experiments in zebrafish. During formation of the Kupffer’s vesicle (KV), the MBs are positioned at the center of a cellular rosette and appear to serve as a scaffold for apical polarity components essential for establishing the lumen. Supporting this idea, when abscission completion was experimentally disrupted, lumens were smaller or failed to form [56•].

Additionally, recent work shows that established polarized epithelia coordinate the positioning of abscission and the midbody with apical adhesive junctions in order to maintain polarity. Epithelial cells must ensure that when they undergo mitosis and cytokinesis, both daughter cells inherit apical membrane and cell junctions, while not creating holes in the membrane. To accomplish this, asymmetric cleavage furrowing and positioning of the MB appears to be essential [57, 58]. In *Xenopus* gastrula embryos, after asymmetric cleavage furrowing completes and the midbody is established, new tri-cellular tight junctions are formed basal to and on either end of the MB [59]. In *Drosophila* sensory organ precursor cell divisions, the new apical junction forms prior to abscission near the midbody [60]. In *Drosophila* ovary follicular epithelium, when apical junction proteins were experimentally mislocalized on baso-lateral membrane, midbodies formed more basally and epithelial invaginations were observed [61].

**Fig. 3:**
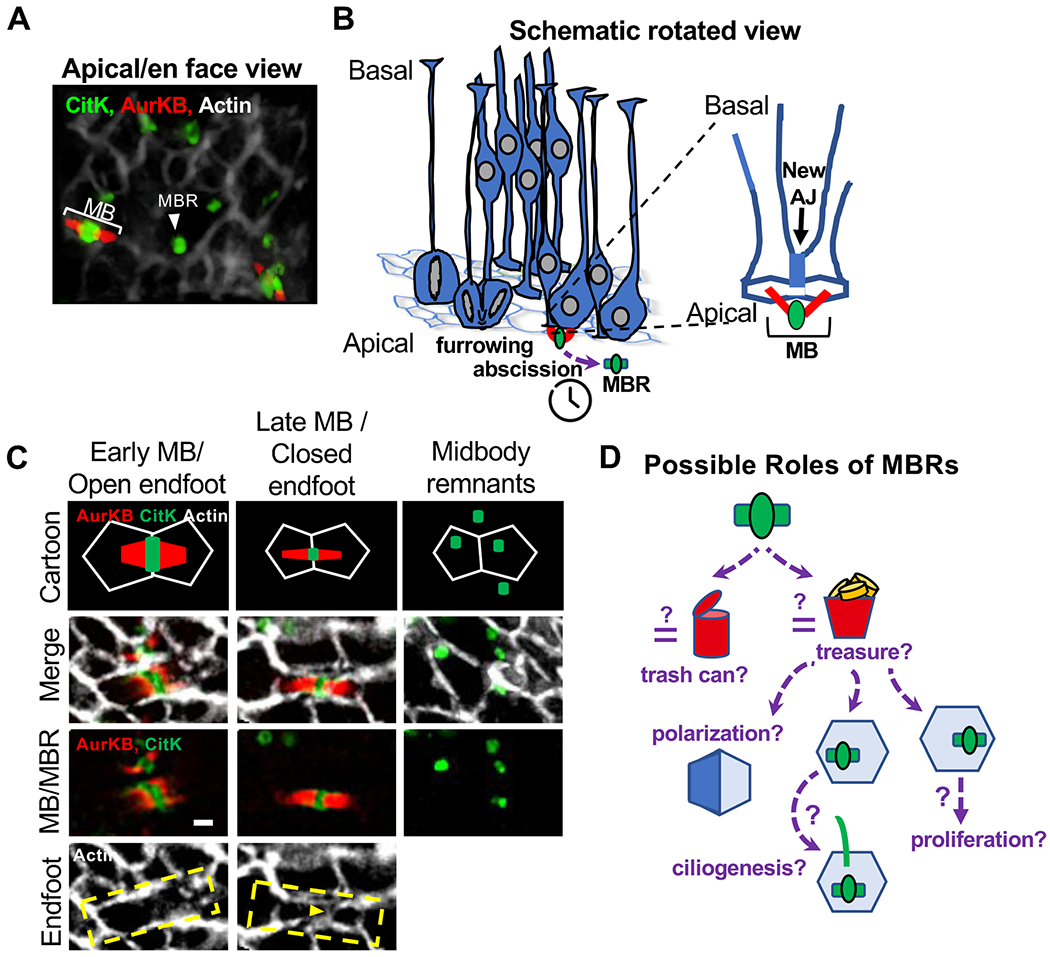
NSC cytokinetic abscission is coordinated with apical membrane segregation and signaling events. (A) En face view of E11 apical membrane where NSC midbodies (MB) form and midbody remnants (MBRs) are deposited. Cortical slab is labeled with phalloidin (actin, apical junctions, AJ), AurKB (MB flanks), and citron kinase, CitK (central bulge and MBRs). (B) Schematic of NSC cytokinesis at the apical membrane. Zoomed view of a pair of daughter cells connected by a midbody and newly forming AJ. (C) MB maturation coordinates with apical endfoot cleavage and new AJ formation. Early midbodies are wider and surrounded by the “open” NSC endfoot. Late midbody is thinner and the endfoot is “closed,” split in two by a new junction (yellow arrowhead) forming between the daughter cells, basal to the MB. MBRs are released at the apical membrane after abscission of both midbody flanks. Scale bar 1 μm. (D) Schematic of proposed roles of MBRs. The MBR could function as a “trash can” to remove unwanted proteins from the newborn daughter cells, or it could be a “treasure chest” for inducing polarization or promoting ciliogenesis or proliferation. See [65••] for methods.

The relationship between apical junctions and midbody positioning has not been directly tested in the developing mammalian brain. In the mammalian neuroepithelium, the apical membrane is the site of polarity cues, cell-cell junctions, and cilia formation. As NSCs go through the cell cycle and divide, they must maintain their apical membrane attachment and re-grow their cilia after each mitosis. In the developing mouse brain, we and others have shown that midbodies align parallel to the apical membrane [21, 62] (Figure 3A, B). When abscission of cortical NSCs occurs and the apical endfoot is split between the two daughter cells, a new adhesive junction is built between the daughter cells, basal to the midbody (Figure 3B, C). The apical junctions appear to surround the two flanks of the NSC midbody (Figure 3C). The midbody may interact with nearby candidate fate determinants like apical par complex, Notch/Numb, and centrosomes [59, 62–64]. Interestingly, in one of the abscission mutants, *Kif20B*, some midbodies are not aligned to the apical membrane [21]. The precise cause of this defect is unclear but could result from a defect in linking the midbody to the apical adhesive junctions, or from premature abscission [65••] before the new apical junctions have fully formed. Following abscission, the midbody remnants remain at the apical membrane or are released into the ventricle [62, 65]. The placement of both the MB and MBR of NSCs highlights how the spatial localization of abscission may be necessary for downstream cell processes. How the mammalian NSC MB forms in the apical membrane and interacts with cell junctions, and how this is remodeled during abscission is not well understood. To date, none of the mouse abscission mutants has reported apical junction defects, but this has not been addressed directly.

## Abscission Duration May Regulate Stemness Versus Differentiation

A major question in developmental and stem cell biology is how do stem cells maintain their potency early in development, but also give rise to daughter cells that differentiate and adopt different fates? Fate specification of daughter cells occurs in the same timeframe as abscission in many systems. In some types of stem cells, daughter cells choose their fate during a “commitment window” in G1 phase, in which they may be receptive to extrinsic signals; G1 phase is also when abscission happens [2, 66]. In *Drosophila* sensory organ development, binary fate decisions between sister cells are made through Notch signaling at their new cell junction while abscission is proceeding [60]. When a neural stem cell divides, each daughter cell can remain stem-like (self-renew), become an intermediate progenitor, or terminally differentiate into a neuron. In mammalian embryonic cortex, there is a time window of a few hours after mitosis when this cell fate decision is plastic [67]. Since abscission occurs in this time window, in close proximity to fate-signals at the apical membrane, it is plausible that abscission duration could influence the reception of these signals.

As new studies of abscission in different stem cell types in vitro and in vivo are published, a common feature is emerging that early development and stemness are associated with longer duration of abscission than later stages of development. Table 2 shows the duration of abscission, the time between midbody formation and midbody severing, measured in different cell types. In a cultured cancer cell line (HeLa), abscission takes about an hour, but it can be delayed if the cell is under certain kinds of stress [68]. By contrast, a mouse embryonic stem cell line shows developmental regulation of abscission duration. Naïve pluripotent cells took an average of 8 h to complete abscission, but those exiting from naïve pluripotency took only about half the time to abscise [69••]. Further, delaying abscission by knocking down *Alix* or *Cep55* resulted in an increase in colony formation, suggesting that increased abscission duration helps stem cells retain potency [69••]. In vivo studies provide more evidence. In *Drosophila* germline stem cell divisions, abscission timing is tightly controlled, and blocking abscission results in mixed daughter fates [70]. In the earliest mouse embryos (4–8-cell stage), the cells are connected by intercellular bridges for extended periods of time (~ 9 h) [71]. Similarly, in zebrafish early embryos, for the first five cell cycles, abscission does not occur and the cells remain connected by intercellular bridges. In the 7th cell cycle, abscission starts to occur with a duration of 40 min, but this decreases to 20 min by cell cycle 13 [72]. Taken together, these findings from early embryos suggest that delaying stem cell abscission may help maintain their potency or coordinate lineages.

## Regulation of Abscission Duration May Be Important for Mammalian Brain Development

Since data from other systems suggest that early high-potency stem cells have longer abscission duration than later less potent stem cells, we wondered whether abscission duration changes in the NSCs of the developing mammalian brain as development proceeds. Between E11 and E13, NSCs in the developing cortex change their mode of division and the types of daughter cells they can produce. At E11, NSCs usually undergo symmetric proliferative divisions, making two daughter stem cells, whereas at E13, NSCs increase neurogenic divisions, producing some daughter cells that stop dividing to differentiate into neurons. Interestingly, in intact explants of the developing cortex, we found that the average duration of abscission decreased between E11 and E13 [65••]. This developmental regulation is consistent with the idea that the timing of abscission is important for stem cell potency and daughter cell fates.

To test this idea further, we studied two mouse mutants in genes that regulate abscission duration, *Kif20B* and *Cep55*. We found that NSC daughter cell fates and brain growth were dramatically disrupted in both mutants. Kif20B is a kinesin-6 family member that localizes to the midbody flanks and constriction sites. When KIF20B is knocked down in a human cancer cell line (HeLa), abscission duration is dysregulated, but still completed [36]. In the brains of mutant mice lacking Kif20B, NSC abscission in the embryonic cortex is faster than in control brains at both E11 and E13. In fact, abscission duration in the mutant mice is almost identical between the two ages (~ 38 min), losing the developmental regulation seen in wild-type brains [65••]. Although this appears to be a modest disruption of abscission timing at the single cell level, there is a profound effect on brain size. This is primarily due to p53-mediated apoptosis in some early NSCs that is associated with abscission [21, 46]. In addition, direct examination of E11 NSC daughter cell fates shows that there is a significant loss of stem cell daughters and concomitant gain in neuron daughters [65••]. Therefore, it is an attractive hypothesis that faster abscission promotes excessive differentiation of daughter cells into neurons in this mutant, depleting the stem cells (and precluding their future progeny).

While Kif20B regulation of abscission duration is subtle, Cep55 loss has a more dramatic effect on abscission. Cep55 localizes to the central bulge of the midbody and recruits ESCRT and ESCRT accessory proteins for abscission completion [73, 74]. When CEP55 is knocked down in HeLa cells, abscission is significantly delayed, and almost all cells fail to abscise, eventually regressing the intercellular bridge and becoming binucleate [75]. Human *CEP55* mutations are associated with a series of human developmental syndromes including stillbirths with hydranencephaly in the most severe cases [18, 20, 30]. Surprisingly, despite the severity of human disease and cell line data, the *Cep55* knockout mouse mutant is born in normal Mendelian ratios and survives to weaning [24•, 29•]. In the knockout, NSC abscission is significantly slower than control NSC abscission but not delayed to the same degree as in cell lines [29•]. NSC abscission usually succeeds, but also fails in ~ 25% of divisions, producing binucleate stem cells and neurons [29•]. Overall, the *Cep55* knockout mouse has a small brain due primarily to increased NSC apoptosis, but also premature neurogenesis [29•]. Interestingly, the apoptosis is dependent on p53, but the premature neurogenesis is not [29•]. This is different than in cell line studies, where p53-mediated cell cycle arrest follows cytokinesis failure (cleavage furrow regression) [76]. It appears that NSCs have a non-p53 pathway that promotes NSC daughter cell differentiation into neurons when abscission is perturbed [46]. It is unknown if this pathway is activated by abscission duration or by proteins in the midbody, directly or indirectly.

## Midbody Remnants: Trash Can or Treasure?

Once abscission has occurred, the post-abscission midbody is referred to as the midbody remnant (MBR). The completion of abscission on one or both flanks of the midbody determines whether the MBR is directly inherited by one daughter cell, or released into extracellular space. If it is released, the MBR can be internalized by one of the daughter cells or neighbor cells [11, 14]. Proteomics and lipidomics approaches have revealed that the MBR contains over 400 proteins and is enriched for certain lipids [3••, 4, 77, 78]. This raises the question: is the cell wasting all this material, or does the MBR serve a role, as either a trash receptacle or a treasure chest (Figure 3D)?

## Abscission: Bilateral or Unilateral?

Recruitment and assembly of the abscission machinery within the midbody is temporally and spatially regulated [10]; however, regulation of abscission on one flank (unilateral) or both flanks of the MB (bilateral) across development and model systems is not well understood. It is plausible that bilateral or unilateral abscission could influence the symmetry of divisions and the resulting daughter cell fate. In MDCK cells cultured as a 2-D monolayer polarized epithelium, some MBRs were observed to be attached to cells by a very thin plasma membrane tether, as seen by scanning electron microscopy [79]. It is not clear whether this tether is long-lasting, or represents an intermediate stage of abscission when only one flank has been cut. In a different scanning electron microscopy study using HeLa cells, this membrane tether was not observed [11]. In other reports in HeLa, dissociated MDCK, and *C. elegans* embryonic cells, the majority of cells completed bilateral abscission [10, 11, 14, 36, 80–82].

Interestingly, in NSCs, we find that bilateral abscission is observable in most divisions regardless of developmental age (E11 and E13) or duration of abscission [65••]. It is possible that unilateral abscission occurs at a later stage of brain development, but we were unable to assay this due to tissue thickness. The presence of thin membrane tethers to MBRs, as was seen in MDCK monolayers, has not been ruled out in NSCs.

## Midbody Remnants May Influence Daughter Cell Fate

As we discussed previously, MBRs from other cell types have the ability to influence polarity from both within the cell or from the cell surface. There is also evidence for MBR influence on cell fate. In different cell types examined so far, MBRs are sometimes associated with differentiation and sometimes with proliferation. In the *Drosophila* germline stem cells, MBR inheritance is stereotyped, and blocking abscission results in mixed daughter cell fates [70, 83]. In the *C. elegans* early embryo, the MBR formed by the first cell division is retained in the posterior daughter, ablating it disrupts embryogenesis, and MBR inheritance is stereotyped in the lineage [15]. Later in *C. elegans* development, in L1 larvae, the Q neuroblast has stereotyped asymmetric divisions that result in three neurons and two apoptotic cells. All of the MBRs from these divisions are released extracellularly and engulfed by a specific neighboring cell that is also responsible for clearing apoptotic cells [14]. These data are consistent with the idea that MBR disposal is regulated developmentally, and that the MBR is shed or degraded by differentiating daughter cells.

Once the midbody is released extracellularly, is it engulfed because it is treasure, or a trash can needing to be degraded? Multiple groups have shown that cancer cells release MBRs and then proceed to engulf and accumulate MBRs at a higher rate compared to stem cells or other cell lines [11, 12, 16, 84]. Interestingly, the accumulation of engulfed MBRs in cancer cells lines is enabled by MBRs’ ability to avoid the lysosome, perhaps by the membrane-bounded MBR coating with actin patches [12]. Limited work has been done on the downstream effect of engulfing midbodies. In HeLa, cells containing MBRs increased transcriptional activity that promotes cell proliferation compared to HeLa cells without MBRs [12]. This is evidence that MBRs could directly influence the ability of a cell to proliferate. MBRs could also be a trash can for stem cells to eliminate damaged proteins or to remove differentiation factors, and therefore would need to be engulfed and degraded. More work on downstream effects of engulfing midbodies across cell types is needed.

Do MBRs influence cell fate in NSC divisions in developing brain? The answer is not known, but there are some suggestive correlations. As mentioned previously, NSC MBRs are deposited at the apical membrane, where many fate-signaling events occur. We showed that MBRs were more abundant on the apical membranes of early brains, when NSC divisions are more symmetric proliferative, compared to later ages, when NSC divisions are more asymmetric and neurogenic [65••]. Additionally, in E11 NSC divisions in culture, MBRs are more likely to be associated with proliferative divisions than neurogenic divisions. These differences suggest developmental regulation of MBR release (bilateral vs unilateral), adhesion to membrane, or engulfment/degradation pathways. It is unknown whether NSCs internalize MBRs. To date, the only mouse mutant that affects the abundance of MBRs in the developing brain is the *Cep55* knockout. Despite some NSCs failing at cytokinesis and becoming binucleate, there are increased MBRs on the apical membrane, regardless of developmental stage [29•]. This could be a manifestation of the delayed abscission found in the *Cep55* mutant NSCs. Alternatively, it could be due to defective MBR disposal or degradation.

## Midbody Remnants May Influence Ciliogenesis

NSCs in the cortical neuroepithelium each have a primary cilium on their apical membrane that serves as an antenna to receive signals from the cerebrospinal fluid, and best known to mediate sonic hedgehog (Shh) signaling [85]. Each cell disassembles its cilium as it prepares for mitosis, and regrows it after mitosis, during the same time window as abscission occurs. This raises the question, do midbodies or MBRs influence cilia growth? There is evidence from MDCK cells cultured as a monolayer polarized epithelium that MBRs enhance ciliogenesis. Following abscission of MDCK cells, MBRs move along the outside of the apical membrane toward the centrosome at the cell apex [13]. It was proposed that the MBR delivers a special membrane patch to the centrosome, and once this interaction happens, the cell begins to grow its cilium [86, 87]. Physically or genetically removing MBRs from the surface reduced the percentage of the MDCK cells developing a primary cilium [13, 79]. These data suggest that, at least in this cell type, the MBR promotes ciliogenesis, perhaps by direct contact. It remains to be seen whether this is also true in other epithelia.

The MBR has been implicated in cell fate and establishment of polarity. While it is clear that there are cell-type differences, the MBR is emerging as an important signaling component. The MBR could serve as a “trash can” receptacle to sequester differentiation-promoting factors, and release of the MBR could be essential to keep stemness and prevent differentiation. In addition or alternatively, the MBR may be a “treasure chest” that promotes proliferation or ciliogenesis (Figure 3D). These roles or MBR contents may be adapted in different cell types or at different developmental stages. More work is needed to understand the importance of the MBR during cortical development and in other stem cells in different developmental contexts.

## Conclusion

Stem cells have special requirements for cell division and cytokinesis, to maintain stemness as they proliferate and then to produce various types of daughter cells during development. Neural stem cells of the embryonic mammalian brain are highly polarized with tiny apical membranes and divide both symmetrically and asymmetrically to produce billions of daughter cells in a short time. This may be why mutations in abscission genes affect brain development more severely than other tissues. These mouse mutant studies, along with evidence from simpler systems, suggest that regulation of midbody positioning in relation to apical junctions, abscission duration, and MBRs contribute to brain development. The mechanisms of how these aspects of abscission are controlled and how they may affect the balance of proliferation versus differentiation are only beginning to be elucidated. Much remains to be learned about how NSCs and other stem cells regulate these different aspects of abscission in order to build polarized tissue structure and give rise to the right daughter cells at the right times.

## Figures and Tables

**Table 1:** Abscission gene mutations cause microcephaly and other phenotypes in mouse and human.

Gene	*Cep55*	*Kif20A (MKLP2)*	*Kif20B*	*Kif14*	*Citron Kinase (CitK)*	*Sept7*
**Abscission function**	Recruit ESCRTs	Localize AurKB to MB	MT bundling	Localize CitK to MB	Maintain MB stability, Localize Kif14	Maintain furrow stability
**Midbody localization**	Central bulge, MBR	Flanks	Early MB: Flanks Late MB: CS	Central bulge, MBR	Central bulge, MBR	Central bulge, MBR?
**NSC midbody Phenotype**	Shorter, not misaligned	Unknown	Wider, misaligned	Unknown	Unknown	Unknown
**Cortical thickness**	↓	↓↓	↓↓	↓	↓	↓↓
**NSC abscission Duration**	Longer	Unknown	Shorter	Unknown	Patient-derived NPCs: longer duration	Unknown
**Binucleate cells**	Yes (mouse, human)	No	No	Unknown	Yes (mouse, rat, human)	No
**Cell cycle exit**	E12.5: ↑	E15.5: ↑	Unknown	Unknown	Unknown	E15.5: ↑
**Apoptosis**	↑↑	↑	↑	↑↑	↑↑	↑↑
**Human syndromes**	mcph, variable MCD, ID/DD, abnormal muscle tone, digits, kidney	Unknown	Unknown	mcph, variable MCD, ID/DD, abnormal digit, eye, kidney	mcph, variable MCD, ID/DD, short stature, abnormal muscle tone, kidney	Unknown
**Refs**	18, 20, 24, 29–31	28	21, 65	25–27, 32	22, 33–34	23, 35

Abbreviations: *MB* midbody, *MBR* midbody remnant *CS* constriction site, *AurKB* Aurora kinase B, *mcph* microcephaly, *MCD* malformations of cortical development, *ID/DD* intellectual disability/developmental delay

**Table 2:** Abscission duration varies in cell types and species and may decrease as differentiation proceeds. All times are means except times between 1st and 2nd abscissions which are medians. Note that comparisons are approximate since different methods of imaging were used.

	Cell type	Measurement	Duration		Refs
			Time to 1st abscission	Time between 1st and 2nd abscission	
**Cell lines in vitro**	HeLa	Furrow ingression to MT disassembly	49– 68 min	Median: 7.5–14.5 min	10, 36
	NS5 (mouse NSC cell line)	Arbitrary point in telophase to MT disassembly	100 min	avg 40min	16
	MDCK (dissociated)	Anaphase to MT disassembly	2 h	avg 20min	80
		MB formation to MT disassembly	90 min	?	55
	mouse ES cell line	MB formation to MT disassembly	naïve pluripotent: 8+ h pluripotency exit: ~4 h	?	69
	*Drosophila* epithelium (notum)	Anaphase onset to MT disassembly	MT disassembly at 42 min. Diffusion up to 5 h	No abscission on 2^nd^ flank	58
**Tissues in vivo**	Zebrafish Blastula	MB formation to MBR release	Cell cycle 7: 40 min Cell cycle 12: ~ 20 min	?	72
	*C. elegans* first cell division	Furrow ingression to MT disassembly	~6.5 min	?	81
	mouse NSCs in embryonic cortex explant	MB formation to MT disassembly	E11.5: 57 min E13.5: 47 min	Medians: E11.5: 30 min E13.5: 15 min	65

Abbreviations are as follows: *MT* microtubules, *MB* midbody, *avg* average, *h* hours, *min* minutes
